# Hierarchical Sub-Pixel Anomaly Detection Framework for Hyperspectral Imagery

**DOI:** 10.3390/s18113662

**Published:** 2018-10-28

**Authors:** Wenzheng Wang, Baojun Zhao, Fan Feng, Jinghong Nan, Cheng Li

**Affiliations:** 1 School of Information and Electronics, Beijing Institute of Technology, Beijing 100081, China; zbj@bit.edu.cn (B.Z.); fengfan@bit.edu.cn (F.F.); jhnan@bit.edu.cn (J.N.); licheng@bit.edu.cn (C.L.); 2 Beijing Key Laboratory of Embedded Real-time Information Processing Technology, Beijing Institute of Technology, Beijing 100081, China

**Keywords:** hyperspectral image (HSI) analysis, anomaly detection, RX, hierarchical structure

## Abstract

Anomaly detection is an important task in hyperspectral processing. Some previous works, based on statistical information, focus on Reed-Xiaoli (RX), as it is one of the most classical and commonly used methods. However, its performance tends to be affected when anomaly target size is smaller than spatial resolution. Those sub-pixel anomaly target spectra are usually much similar with background spectra, and may results in false alarm for traditional RX method. To address this issue, this paper proposes a hierarchical RX (H-RX) anomaly detection framework to enhance the performance. The proposed H-RX method consists of several different layers of original RX anomaly detector. In each layer, the RX’s output of each pixel is restrained by a nonlinear function and then imposed as a coefficient on its spectrum for the next iteration. Furthermore, we design a spatial regularization layer to enhance the sub-pixel anomaly detection performance. To better illustrate the hierarchical framework, we provide a theoretical explanation of the hierarchical background spectra restraint and regularization process. Extensive experiments on three hyperspectral images illustrate that the proposed anomaly detection algorithm outperforms the original RX algorithm and some other classical methods.

## 1. Introduction

Hyperspectral imagery (HSI) records detailed spectral information in hundreds of narrow contiguous bands over the visible light spectrum to the Near Infrared (NIR) or Short-Wave Infrared (SWIR) bands. Each HSI pixel is a vector that represents the radiance or reflectance value of each band; therefore, HIS can be considered a 3-D cube. Such data provide deterministic spectral information about different materials and objects in the scene. Benefitting from this unique characteristic, HSI is becoming a valuable tool for many real-world applications, such as agriculture surveillance [1,2], mineralogy [3], and environmental sciences [4,5,6]. Among these applications, hyperspectral image anomaly detection plays an important role.

Anomaly detection in HSI can be regarded as a particular case of target detection that assumes no prior knowledge of the target’s spectral signature [7]. The main task of anomaly detection is to detect an object with an unusual spectral signature with respect to the background, especially small man-made objects. Over the past two decades, several algorithms for hyperspectral anomaly detection have been proposed, such as the RX detector (RXD) [8], robust PCA (RPCA) [9], low-rank and sparse representation (LRASR) [10], and low-rank and sparse matrix decomposition-based Mahalanobis distance (LSMAD) [11]. These algorithms can be classified into two classes: Statistical-based methods and low-rank or sparse matrix decomposition-based methods.

The most widely used statistical-based anomaly detection method is the RX anomaly detection algorithm, which is also considered a benchmark among most classical anomaly detection algorithms [7]. The RX anomaly detection algorithm is based on the assumption that the HSI can be modeled as a multivariate Gaussian distribution in which the Mahalanobis distance between the pixel under test and the pixels within the local or global background has been calculated. However, real-world HSIs hardly follow the Gaussian assumption, since the noise and background elements in actual scenes are complicated and pose modeling difficulties. To overcome these limitations, improved RX-based algorithms have been proposed for better local or global background estimation, including local RX algorithms [12,13] and kernel-based RX algorithms [14,15].

Another anomaly detection class consists of low-rank or sparse matrix decomposition-based methods. The RPCA method separates the original data matrix into a low-rank matrix and a sparse matrix by two simple constraints. The LSMAD method combines the RPCA and RXD, and then obtains a low-rank background matrix and calculates the Mahalanobis distance according to the obtained low-rank components. For the LRASR anomaly detection method [10], a background dictionary is constructed, although it may vary in different experiments, which leads to unstable detection results. However, these algorithms employ knowledge of both background and anomalies; thus, they can improve the detection performance to some extent.

In this paper, instead of adding or improving sparse constraints directly as in LRASR and RPCA, we build a new hierarchical architecture to restrain the background spectra while preserving the anomaly target spectra. A simple and effective method, i.e., the hierarchical-RX (H-RX) method, is proposed for improving the performance of classical RX algorithms and achieving better anomaly detection performance, especially for sub-pixel anomaly detection tasks.

Since the classical RX anomaly detector in certain situations cannot accurately accentuate the anomaly targets and restrain the backgrounds, we use a hierarchical framework to solve this problem by processing the hyperspectral data with several layers, and the RX anomaly detectors of different layers are linked in series. After each layer’s anomaly detection, a nonlinear function is used to restrain the background spectra based on the results of the RX detector. Then, the adjusted spectral data are organized and sent to the next layer until the RX detector’s result meets the iteration stop condition. Restraining the background of spectral components facilitates modeling their distribution and leads to more significant anomaly spectra. In this approach, the RX performance is gradually enhanced layer by layer. 

The contributions of our work are summarized as follows.

(1) A novel hierarchical framework for I anomaly detection is proposed. It restrains the background spectra and highlights the anomalies by multilevel processing.

(2) We prove the rationality of the proposed method theoretically. After several layers of background restraints, the anomaly detection performance is gradually enhanced in theory, especially for sub-pixel anomaly detection tasks.

(3) Experimental results on three hyperspectral images show that the proposed method significantly enhances the traditional RX method and outperforms the other improved classical RX-based anomaly detection algorithms.

The remainder of this paper is organized as follows. Section 2 reviews the original RX detector method and the detection characteristics of the RX algorithm and proposes our hierarchical suppression anomaly detection method. Section 3 provides theoretical analyses of our method. In Section 4 presents the experimental results, and Section 5 describes the conclusions.

## 2. Related Work and Proposed Method

In this section, we first review the original RX anomaly detection algorithm. Then, we propose the hierarchical-RX method.

### 2.1. Brief Introduction to the RX Algorithm

The RX anomaly detection algorithm is one of the most fundamental and popular algorithms for anomaly detection tasks, and it has been considered the benchmark of anomaly detection fIHSI [7]. The RX algorithm is a constant false alarm rate (CFAR) adaptive anomaly detector derived from the generalized likelihood ratio test [8].

In the RX algorithm, anomaly detection is formulated as two hypotheses, $H_{0}$ and $H_{1}$. The first hypothesis, $H_{0}$, models the background as a Gaussian distribution with a zero mean and an unknown background covariance matrix that is estimated locally or globally from the data. The second hypothesis, $H_{1}$, models the target as a linear combination of a target signature and background noise. Therefore, under $H_{1}$ and $H_{0}$, spectral vectors are represented by a Gaussian distribution with a mean equal to the signature of the target and an additive noise equal to the background covariance matrix, respectively. Consider I-D HSI cube with A rows, B lines and L spectral bands, where:

(1) A∗B=N 

Therefore, all spectra of this hyperspectral image can be arranged in an L×N matrix as $\;X=\left[x_{1},x_{2},\cdots ,x_{n}\right]$. The two competing hypotheses that the RX algorithm must distinguish are given as follows:

(2) $$\begin{array}{ll} H_{0}:x=n & \;(\mathrm{Target}\;\mathrm{absent})\\ H_{1}:x=a*s+n & \;(\mathrm{Target}\;\mathrm{present}) \end{array}$$

where a=0 under $H_{0}$ and a>0 under $H_{1}$. n is a vector that represents the background clutter noise process, and s is the spectra signature of the signal (target) given by $s=\left[s_{1},s_{2},\cdots ,s_{j}\right]$. Therefore, the RX algorithm anomaly detection can be defined as follows:

(3) $$RX(r)={\left(r-\mu_{b}\right)}^{T}{\left(\frac{M}{M+1}C_{b}+\frac{1}{M+1}(r-\mu_{b}){\left(r-\mu_{b}\right)}^{T}\right)}^{-1}(r-\mu_{b})\left\{ \begin{array}{l} \geq \lambda \;\mathrm{Target}\\ <\lambda \;\mathrm{Background} \end{array}\right..$$

In addition, this formula can be simply defined as:

(4) $$\delta_{rx}(r_{ij})={\left(r_{ij}-\mu_{b}\right)}^{T}C_{b}^{-1}(r_{ij}-\mu_{b}),$$

where $\mu_{b}$ represents the estimated mean spectral signature and

(5) $$C_{b}=\frac{1}{Q}\sum_{i=1}^{Q}(x(i)-\mu_{b}){\left(x(i)-\mu_{b}\right)}^{T}.$$

Here, $C_{b}$ is the estimated covariance matrix of the N pixels belonging to the background. The RX algorithm is based on exploiting the difference between the spectral signatures of an input pixel with its surrounding neighbors. This distance comparison is very similar to the Mahalanobis distance measure [7].

Due to the equivalence mentioned above, influence factors on the RX performance can be identified from the perspective of Mahalanobis metrics. The larger Mahalanobis distance indicates the spectra belongs to anomaly category with a higher probability. That is, it greatly depends on the dissimilarity between the anomaly spectra and the average one. Considering the facts that anomaly spectra are sparse among background, and background spectra are not always same, since of the same object with different spectra phenomenon, we can thus infer that the metric is affected by two aspects: (1) Difference between anomaly and average spectra; (2) variation of background spectra.

Such kind of factors would have more impact on the sub-pixel anomaly detection with small ratio. In general, it is intractable to discriminate legitimate anomalies from detections that are not of interest, since of the deficiency of prior knowledge on the anomaly type [7]. Specifically, when the ratio of sub-pixel is small so that anomaly spectra is much similar to the background one, the Mahalanobis distance of false anomaly may be larger than one of legitimate anomalies, and thus degrade the robustness of RX detector.

Given aforementioned analysis, one of feasible strategies to improve the original RX is enhancing discriminability between anomaly spectra and background ones. This is also the motivation of the proposed hierarchical anomaly detection framework. But unlike the iterative methods [16,17], which consist of the many layers with same affects and functions, we built the framework by using different layers with different purposes. Moreover, they use iterative method to remove the spectra once it determined as anomalies. Different from them, we design the hierarchical framework, where we just restrain the background spectra by a nonlinear suppression function so as to better estimate the mean background vector and enhance background-anomalies contrast.

### 2.2. Hierarchical-RX Algorithm

In this paper, we propose that a restraint on the background spectra is helpful for anomaly detection problems. In addition, regularization on the anomaly detection result is helpful for pixel/sub-pixel anomaly detection tasks. The major characteristics of the proposed hierarchical framework can be summarized as follows.

(1) The original RX detector can be considered a single-layer detector, whereas the proposed H-RX detector consists of several layers of traditional RX detectors, and each layer is linked in series.

(2) After each layer of detection, the background spectra are restrained based on the current layer’s anomaly score. In this approach, anomaly spectra become more distinguished from the background spectra, which implies that the H-RX method can achieve a better performance in the same situations to which the original RX detector is applied. 

(3) To enhance the result for pixel/sub-pixel anomaly detection, a regularization method based on point spread characteristics is designed to eliminate the effects between the anomaly spectra and background spectra around anomaly pixels.

**Figure 1:**
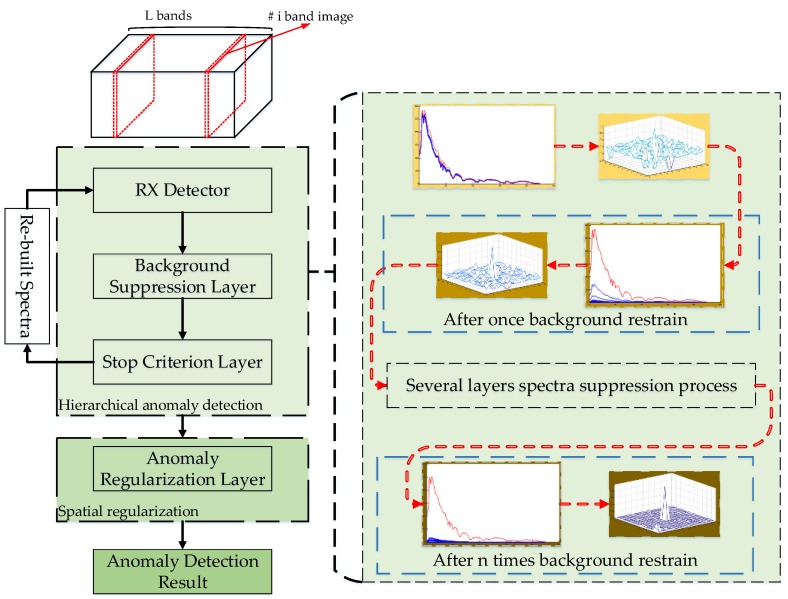
Framework of the proposed hierarchical Reed-Xiaoli (H-RX) anomaly detection algorithm.

The flowchart of the proposed hierarchical-RX anomaly detection method is shown in Figure 1. Compared with the original RX detection method, the H-RX anomaly detection architecture is structurally divided into two phases: (1) Hierarchical anomaly detection, and (2) anomaly detection result regularization in spatial domain.

For the former phase, a background suppression layer is developed to restrain the background spectra of the input hyperspectral data and a stop criterion layer is designed to determine whether the data need further detection processing. For the anomaly detection result regularization phase, the result regularization layer is tactically designed based on the point spread characteristics, and it is designed to enhance the anomaly detection result.

Consider HSI with N pixels and L spectral bands, which are arranged in an L×N matrix as $X=\left[x_{1},x_{2},\cdots ,x_{n}\right]$. For the $k_{th}$ layer, the H-RX detector’s output of this layer can be represented as follows:

(6) $$y^{k}={\left(x{\left(i\right)}^{k}-\mu^{k}\right)}^{T}{\left(C^{k}\right)}^{-1}(x{\left(i\right)}^{k}-\mu^{k}),$$

where $x{\left(i\right)}^{k}$ and $\mu^{k}$ represent the spectral matrix and the mean spectra of the $k_{th}$ layer, respectively; $C^{k}$ represents the correlation matrix of the $k_{th}$ layer; and $y^{k}$ is the output of the $k_{th}$ layer and represents the possibility of that an anomaly target corresponds to the spectra, and it has a value within [0, 1]. Then, each spectral vector $x_{i}^{k}$ is transformed by multiplying a nonnegative number $q\left(y_{i}^{k}\right)$ based on its output score as follows:

(7) $$x_{i}^{k+1}=q(y_{i}^{k})x_{i}^{k}.$$

**Figure 2:**
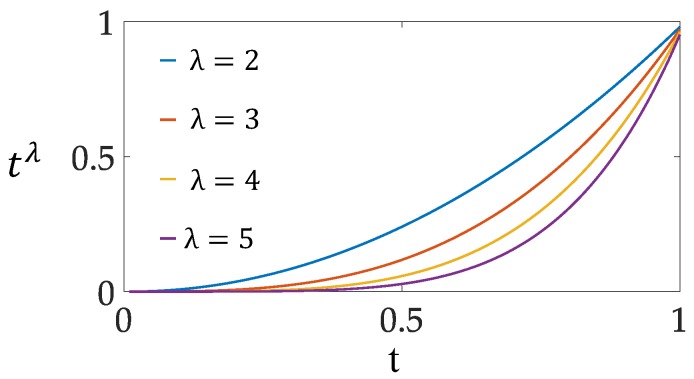
Shape of the nonlinear suppression function with different choices of λ in the background suppression process.

In the formulation, the nonlinear function q(t) is imposed on the spectral vector $x_{i}^{k}$. We consider this function a “soft-threshold” operation that retains the spectrum $x_{i}$, whose output score is large, while suppressing the spectrum $x_{i}$, whose output score is small. In this way, the undesired background spectra are gradually suppressed after each layer’s detection, whereas the target spectra will remain unchanged. In this paper, the nonlinear suppression function is defined as follows:

(8) $$q(t)=\left\{ \begin{array}{l} q(t)=t^{\lambda },\;0\leq t\leq 1\;\\ 0,\;t\text{<}0 \end{array}\right.,$$

where λ is a positive parameter to adjust the shape of the function (8). Figure 2 shows the shape of the function (8) under different choices of λ. 

The potential anomaly spectra and the transformed background spectra will be used to construct the new input data for the anomaly detector in the ${\left(k+1\right)}_{th}$ layer. The aforementioned steps will be repeated until the output $y^{k}$ meets the stop criterion. In this paper, we calculate $\delta_{k}$, which is the error of the average output energy of the current layer and the previous layer, and it can be defined as follows:

(9) $$\delta_{k}=\frac{1}{N}{\left\|y^{k-1}\right\|}_{2}^{2}-\frac{1}{N}{\left\|y^{k}\right\|}_{2}^{2}\left\{ \begin{array}{l} >\varepsilon ,\;\mathrm{Continue}\\ \leq \varepsilon ,\;\mathrm{Stop} \end{array}\right..$$

In the Equation (9), ε refers to a small positive number. Therefore, if $\delta_{k}\leq \varepsilon$, then the iteration will be stopped; whereas if $\delta_{k}>\varepsilon$, then the operation proceeds directly to the next iteration. After a range of anomaly detection, we incorporated a point spread function (PSF) [18,19]-based regularization into our HSI anomaly detection framework to promote the detection results under small anomaly targets.

The spectral mixing model for a sub-pixel target is a difficult and complicated modeling task. We consider spectral mixing to have an effect on the detection result; therefore, we design the regularization method in the spatial domain to enhance the anomaly detection performance. In this regularization method, we modeled the radiation character of the anomaly target by using the PSF as follows:

(10) $$I(x,y)=I_{0}\exp\left(-\frac{1}{2}\left[\frac{{\left(x-x_{0}\right)}^{2}}{\sigma_{x}^{2}}+\frac{{\left(y-y_{0}\right)}^{2}}{\sigma_{y}^{2}}\right]\right).$$

In the Equation (10), $I_{0}$ is a pixel of the H-RX detection result; $\left(x_{0},y_{0}\right)$ represent the location of this pixel; $\sigma_{x}$ and $\sigma_{y}$ are the horizontal and vertical extent parameters, respectively; and I(x,y) denotes the value of the surrounding location (x,y). Then, for the center pixel, we have the point spread indicator p:

(11) $$p=\frac{\ln I_{0}-\ln I_{M}}{\ln I_{0}-\ln I_{N}},$$

where $I_{M}$ and $I_{N}$ represent the average value of the direct neighbor domain and the diagonal neighbor domain, respectively. The point spread indicator p of all pixels in anomaly detection result is calculated. Then, the pixel is a potential anomaly target if the p value is in the protection interval.

At this point, the H-RX is complete. Briefly, the outline of the proposed H-RX algorithm for hyperspectral anomaly detection is given as follows.


**Algorithm 1 Hierarchical-RX Algorithm**
**Input and Initialization:** 1. Spectral matrix $X=\left[x_{1},x_{2},\cdots ,x_{n}\right]$, target spectrum $d\in {\mathbb{R}}^{L\times 1}$, set tolerance ε>0, k=1, $X^{1}=X$**Hierarchical Background Spectral Restraint:** 2. $y^{k}={\left(x{\left(i\right)}^{k}-\mu^{k}\right)}^{T}{\left(C^{k}\right)}^{-1}(x{\left(i\right)}^{k}-\mu^{k})$ 3. $x_{i}^{k+1}=q(y_{i}^{k})x_{i}^{k}$ 4. Rebuild data $X^{k}$ 5. k⇐k+1**Stop Criterion:** 6. $\delta_{k}=\left(1/N\right){\left\|y^{k}\right\|}_{2}^{2}-\left(1/N\right){\left\|y^{k-1}\right\|}_{2}^{2}$; if $\delta_{k}>\varepsilon$, go back to step 2; else, go to step 7 **Anomaly Regularization:** 7. $p=\left(\ln I_{0}-\ln I_{M}\right)/\left(\ln I_{0}-\ln I_{N}\right)$**Output:** 8. Final outputs: $y^{k}=\left[y_{1}^{k},y_{2}^{k},\ldots ,y_{N}^{k}\right]\in {\mathbb{R}}^{1\times N}$.

## 3. Theoretical Analysis

Here, we provide theoretical analyses of the proposed H-RX algorithm. Firstly, a detailed description of our designed background spectral suppression layer is provided. Secondly, the stop criteria layer is analyzed. Finally, the motivation and function of the spatial regularization layer is clarified.

### 3.1. Background Spectral Suppression Layer

Here, we provide a detailed description and theoretical analysis of the background spectral suppression layer. For the ${\left(k+1\right)}_{th}$ layer and the prior $k_{th}$ layer, according to formula (7) and (8), we have

(12) $$x_{i}^{k+1}=\left\{ \begin{array}{cc} {\left(y_{i}^{k}\right)}^{\lambda }x_{i}^{k}, & 0\leq y_{i}^{k}\leq 1\\ 0, & y_{i}^{k}>1 \end{array}\right.\;$$

Because $y_{i}^{k}\in \left[0,\;1\right]$, the detection result between the ${\left(k+1\right)}_{th}$ layer and $k_{th}$ layer has the following relationship:

(13) $$y_{i}^{k+1}=\left\{ \begin{array}{l} y_{i}^{k+1}<y_{i}^{k},0\leq y_{i}^{k}<1\\ y_{i}^{k+1}=y_{i}^{k},y_{i}^{k}=1 \end{array}\right..$$

Based on this relationship, the spectral result between the ${\left(k+1\right)}_{th}$ layer and $k_{th}$ layer has the following relationship:

(14) $$x_{i}^{k+1}-x_{i}^{k}=\left\{ \begin{array}{l} <0,0\leq y_{i}^{k}<1\\ 0,y_{i}^{k}=1 \end{array}\right..$$

For the background spectral results, each spectral vector $x_{i}^{k}$ is multiplied by a number smaller than 1 and every element of this spectral vector is changed to a much smaller value. However, for the anomaly target spectral result, the original value is maintained for every band. Then, the difference between the background spectral result and anomaly target spectral result will be enlarged, and the background spectral result is restrained so that the vector has only a small value (close to a zero vector). 

As a result, the output of the background spectrum will be restrained to a small value, but the output will be much larger for the anomaly spectrum. In the ideal situation,

(15) $$y_{i}^{k}=\left\{ \begin{array}{l} 0,\;\mathrm{background}\\ 1,\;\mathrm{anomaly}\;\mathrm{target} \end{array}\right..$$

After performing background spectral suppression layer processing, we have the following:

(16) $$x_{i}^{k+1}=\left\{ \begin{array}{l} x_{i}^{k},y_{i}^{k}=1\left(x_{i}\;\mathrm{is}\;\mathrm{anomaly}\;\mathrm{target}\;\mathrm{spectral}\right)\\ 0,y_{i}^{k}=0\left(x_{i}\;\mathrm{is}\;\mathrm{background}\;\mathrm{spectral}\right) \end{array}\right..$$

After several layers of restraint operations, the background spectral suppression results in the vector containing only the value 0 and only the anomaly target spectrum retaining its original vector value. 

The background spectral suppression layer has the following characteristics.

(1) The output of the H-RX algorithm will be restrained to a small constant for the background spectrum, whereas this constant will generally retain the original value for the anomaly target spectrum.
(2) This layer can increase the difference between the target spectrum and background spectrum.
(3) Because the background spectrum is restrained to a zero vector, the sparsity of the rebuilt data will be significantly increased.
(4) Although the H-RX algorithm contains several RX detectors, as the data sparsity increases, the calculation speed of each layer with the exception of the first layer will increase.

The abovementioned analysis indicates that the performance of the H-RX detector of the $k_{th}$ layer is equal to or better than that of the ${\left(k+1\right)}_{th}$ layer. By using the background constraint operation, the magnitude of certain background spectra will be suppressed to zero, which leads to an increase of the difference between the background and the target spectra and makes the target easier to detect. The simulation of this situation is shown in Section 4.

### 3.2. Stop Criteria Layer

Based on the analysis of the background spectral suppression layer, we know that after several layer restraint operations, the spectra in the hyperspectral image contains the following characteristics:

(1) The background spectral magnitude is reduced while its direction in the spectral space is maintained, and after several ranges of suppression operations, it will be transformed close to a zero vector.
(2) Concurrently, the target spectra remain unchanged.

Then, after a range of suppression and detection, the spectral vector in the transformed hyperspectral image is almost restrained to a zero vector and only the anomaly target spectra contain the original spectral vector. As a result, the detection results will contain a small value (close to zero) that corresponds to the background spectra, whereas a much larger value is observed for the anomaly target spectra.

In the hierarchical framework, if there is only one anomaly target, the output energy of the current layer can be formulated as follows:

(17) $$y_{Energy}^{k}={\left\|y^{k}\right\|}_{2}^{2}=1+\gamma^{k},$$

where 1 is the energy of the anomaly and $\gamma^{k}$ represents the energy sum of the background. For the next layer, the output energy can be formulated as follows:

(18) $$y_{Energy}^{k-1}={\left\|y^{k}\right\|}_{2}^{2}=1+\gamma^{k-1}.$$

Based on these equations, we can calculate $\delta_{k}$, the error of the average output energy of the current layer and the previous layer, which is defined as follows:

(19) $$\delta_{k}=\frac{1}{N}{\left\|y^{k-1}\right\|}_{2}^{2}-\frac{1}{N}{\left\|y^{k}\right\|}_{2}^{2}.$$

Thus, we have:

(20) $$\delta_{k}=\frac{1}{N}(\gamma^{k}-\gamma^{k-1}).$$

Based on the above analysis, $\gamma^{k}$ and $\gamma^{k-1}$ are small values and are close to zero; then, $\delta_{k}$ will also converge to zero. Therefore,

(21) $$\delta_{k}=\left\{ \begin{array}{ll} \mathrm{go}\;\mathrm{back}\;\mathrm{to}\;\mathrm{background}\;\mathrm{suppression}\;\mathrm{layer}, & \delta_{k}>\varepsilon \\ \mathrm{out}\;\mathrm{put}\;\mathrm{the}\;\mathrm{result}, & \delta_{k}\leq \varepsilon \end{array}\right.\;$$

where ε refers to a small positive value (we set this value equal to $10^{-4}$). By comparing the value between $\delta_{k}$ and ε, the algorithm makes the decision to go directly to the next iteration or stop the iteration detection.

**Figure 3:**
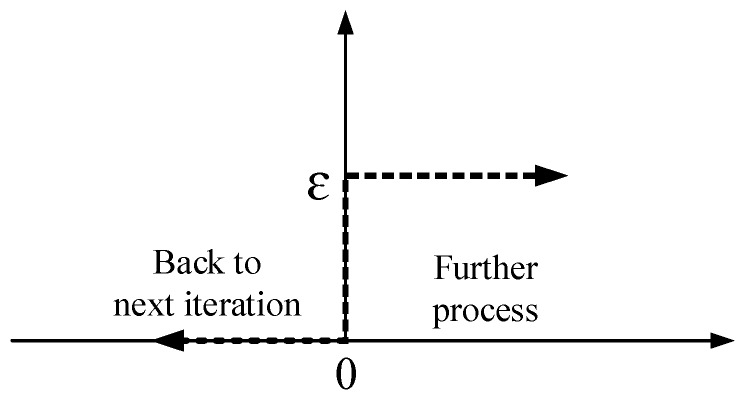
Stop criteria layer model.

Then, the stop criteria layer can be described as a low-pass filter like Figure 3 shown us.

In the whole H-RX algorithm process, the stop criteria later plays the important roles of determining when to stop the detection and identifying whether a potential advantage still exists to improve the anomaly detection performance.

### 3.3. Spatial Regularization Layer Analysis

Before the final detection result is output, we design a regularization layer to enhance the detection performance in the spatial domain. In HSI, the background spectra around the target may be influenced by the target spectra, which prompts consideration of the similarity of the background spectra with the target spectra. As a result, these background pixels have a similar or larger value than the target in the detection result, which leads to false detection. 

In this paper, we focus on achieving a better anomaly detection performance for pixel/sub-pixel anomaly targets. To achieve this goal, the noise caused by pixel-mixing phenomena should first be smoothed. Therefore, the key issue of the filtering module is determining how to distinguish the target and noise. Here, we introduce a key technique in infrared target detection systems reported in the literature [20], and then we design an efficient pixel/sub-pixel regularization filter for the regularization layer.

Based on the assumption that the model of pixel-mixing phenomena influences the detection result, we define the following formula:

(22) $$f_{D}(x,y)=f_{T}(x,y)+f_{B}(x,y)+f_{N}(x,y),$$

where (x,y) represents the pixel location and $f_{D}$, $f_{T}$, $f_{B}$ and $f_{N}$ are the detection result, target, background and noise, respectively. In the method, the radiation character in the detection result caused by pixel mixing has been modeled using the PSF as follows:

(23) $$I(x,y)=I_{0}\exp\left(-\frac{1}{2}\left[\frac{{\left(x-x_{0}\right)}^{2}}{\sigma_{x}^{2}}+\frac{{\left(y-y_{0}\right)}^{2}}{\sigma_{y}^{2}}\right]\right),$$

where $I_{0}$ is the value of the anomaly detection result, $\left(x_{0},y_{0}\right)$ denotes the center location of the candidate target, $\delta_{x}$ and $\delta_{y}$ are the horizontal and vertical extent parameters, respectively, and I(x,y) represents the value of the surrounding location (x,y).

**Figure 4:**
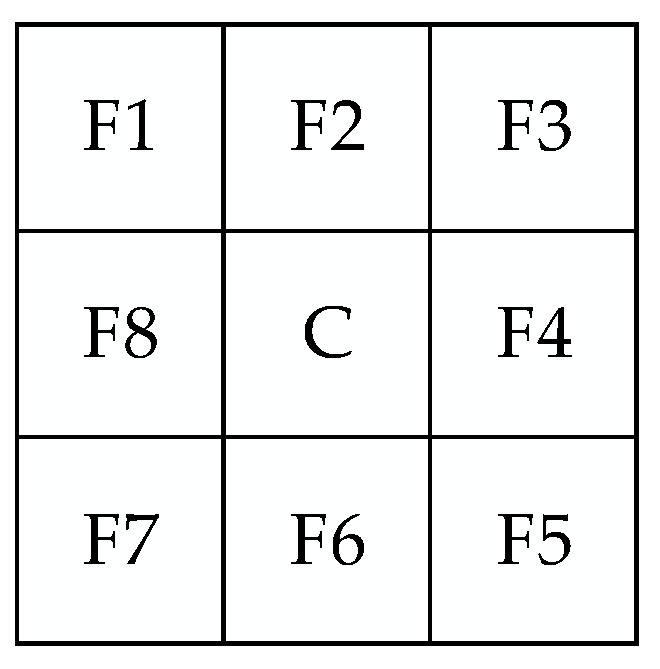
Surrounding structure of the pixel C.

Considering the task of single pixel target detection and the above radiation character of the target, a point spread indicator is proposed to protect the potential target and combined with a median filter to smooth the noise. As shown in Figure 4, suppose there is a center pixel C that is surrounded by the direct neighbor domain $F_{m}$ (m=2, 4, 6, 8) and the diagonal neighbor domain $F_{N}$ (n=1, 3, 5, 7).

In the formulation, $I_{M}$ and $I_{N}$ represent the average value of the direct neighbor domain and the diagonal neighbor domain, respectively. As Equation (23) indicates, the relationship among $I_{M}$, $I_{N}$ and $I_{0}$ can be obtained approximately as follows:

(24) $$I_{M}=I_{0}\exp\left(-\frac{1}{2\sigma_{x}\sigma_{y}}\right).$$

(25) $$I_{N}=I_{0}\exp\left(-\frac{1}{\sigma_{x}\sigma_{y}}\right).$$

Thus, we have the following:

(26) {lnI0IM=12σxσylnI0IN=1σxσy.

If the center pixel is an anomaly target, the value of this pixel and the surroundings will be obtained from Equation (26), whereas a misleading target will not be obtained. To distinguish the target and misleading target effectively, the point spread indicator p is defined as follows:

(27) $$p=\frac{\ln I_{0}-\ln I_{M}}{\ln I_{0}-\ln I_{N}}.$$

The indicator p of the pixel/sub-pixel target is equal to 0.5 only under ideal conditions. Hence, the protection interval of the p value is set as [0.2, 0.8]. By calculating the point spread indicator p of all pixels in the original image, the following condition holds: The pixel is a potential target only if the p value is in the protection interval. The target protection filter strategy combines the anomaly detection result of potential target pixels with the result of a (3×3) or (5×5) median filter of the other pixels. 

## 4. Experimental Results and Analysis

In this section, we evaluate the proposed method on three hyperspectral images. In the experiments, we compare our H-RX algorithm with commonly used anomaly detection algorithms: Original RX, Kernel RX, RPCA and low-rank and sparse representation (LRASR). The former algorithm is a classical anomaly detection algorithm, which is also considered a benchmark among the most classical anomaly detection algorithms. Kernel RX is one of the most famous of the improved algorithms for RX. Another two algorithms are recently proposed low-rank-based anomaly detection algorithm. It may be a stretch to try and draw too much from a comparison of four algorithms and only three images, however, we believe our experiments point to the clear potential of the proposed framework.

To compare the performance of different algorithms, we demonstrate the algorithms based on receiver operating characteristic (ROC) curves, which describe the varying relationship of the detection probability and the false alarm rate and are used to provide performance comparisons of different detectors [21,22]. Based on a ground truth image, the ROC curve expresses the relationship between the false alarm rate $\left(F_{a}\right)$ and the probability of detection $\left(P_{d}\right)$ at different thresholds for the anomaly detector’s output. $F_{a}$ and $P_{d}$ are defined as follows:

(28) $$F_{a}=\frac{N_{f}}{N_{b}},\;P_{d}=\frac{N_{c}}{N_{t}},$$

where, $N_{f}$ represents the number of false alarm pixels, $N_{b}$ is the total number of background pixels, $N_{c}\;$ indicates the number of correct detection target pixels, and $N_{t}$ is the number of total true target pixels. For further comparison of the performance of anomaly detectors, the area under the curve (AUC) [23,24], which is one of the most frequently used performance measures extracted from the ex-ROC, has been used.

### 4.1. Dataset Description

In this paper, we evaluate the performance of the proposed H-RX method on three hyperspectral images. Moreover, for the purpose of verifying the method more objectively and comprehensively, we select datasets that were obtained from different hyperspectral imaging cameras. 

The first two datasets are collected by the Airborne Visible/Infrared Imaging Spectrometer (AVIRIS) over ocean regions [25,26]. The main difference between those datasets is the amount of clouds, which influences background complexity. Those data can be downloaded from NASA’s website (AVIRIS Dataset download website: http://aviris.jpl.nasa.gov/data/get_aviris_data.html.), where the index of these two datasets are f100830t01p00r19 and f131117t01p00r05, respectively. Due to the large size of the original data, we select two images for the experiments. The upper-left corner of each image is (294, 1665) and (330, 7280), respectively.

The third dataset is the most commonly used one for hyperspectral anomaly target detection. It is collected by the Hyperspectral Digital Imagery Collection Experiment (HYDICE) from a land cover region [27], which is mainly composed of buildings, vegetation, road, and vehicles. More details can be found from the website of US Army Corps of Engineers (HYDICE Dataset download website: https://www.erdc.usace.army.mil/Media/Fact-Sheets/.). Here, 162 bands remained after removing the water absorption bands.

**Figure 5:**
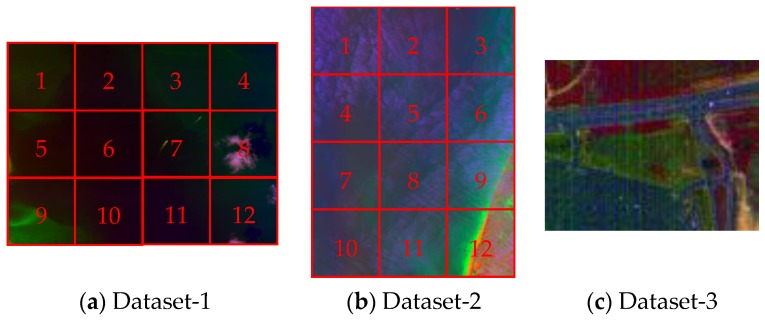
False color image of each dataset.

The descriptions of each dataset are shown in Table 1, and corresponding false color images are displayed in Figure 5. For further experiments, we divide the first and second image into twelve patches, which are indexed by the serial number. One can see that there exists, some ship targets which can be selected to generate the sub-pixel anomalies targets in the number 7 patch of Dataset-1, and number 3 patch of Dataset-2.

### 4.2. Performance Analysis of Background Suppression Layer

The proposed hierarchical anomaly detection algorithm is composed of several layers with different purposes. The most important and novel one is the background suppression layer, which significantly enhances the original RX detection performance. Then, to prove the theory and real effect of this layer, we use a part of the data region in Dataset-1 for a detailed analysis.

**Figure 6:**
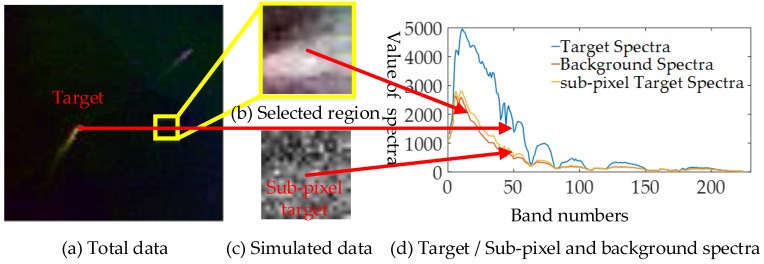
(**a**,**b**) False color image of the total dataset and the selected experimental region data, respectively; (**c**) first band image of the simulated data with the sub-pixel anomaly target; (**d**) target, with the background in location (10, 10) and mixed sub-pixel target spectra.

In this experiment, the ship spectra have been used as the target spectra. We select the seventh patch from the first dataset as the background region (upper-left corner is (201, 101)), the size of the selected background region is (20×20). As shown in Figure 6, we generated the sub-pixel target (abundance ratio equal to 0.1) at the location (10, 10), and then added Gaussian white noise with 30-dB SNR.

Here, we focus on two important and urgent problems that must be resolved to validate the proposed method: (1) Regards the performance of the proposed background suppression layer, (2) considers whether rebuilding the hyperspectral information is useful for enhancing the anomaly detection performance.

For the first question, the difference between the anomaly target and background according to the previously mentioned theoretical analysis will remarkably increase after several background suppression layer processing steps. Then, to verify this phenomenon, the detection results of the original RX with the restraint process applied once and twice were compared directly.

**Figure 7:**
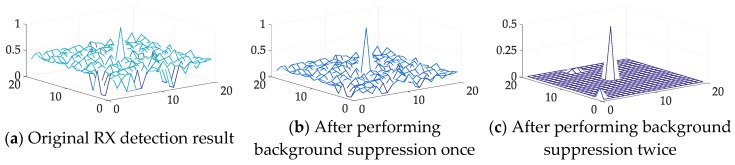
(**a**) 3-D visualization of the original RX detection result; (**b**) 3-D visualization of the result after one background restraint process; and (**c**) 3-D visualization of the result after two background restraint processes. The sub-pixel anomaly target has been gradually increased.

As illustrated in Figure 7, we can see that after one background restraint, the anomaly target is notably different than the original result. Moreover, by suppressing the background once again, the background is restrained to a smaller value and the anomaly target is much more remarkable than the background.

Moreover, to quantitatively analyze the background suppression performance, the distance between the value of the anomaly target and the background with different process steps was also calculated with following formula, and the results are illustrated in Table 2. Note that our method will hold for the situation whether the magnitudes of anomaly target spectra are larger than average ones of background or not.

(29) $$\mathrm{Distance}=\left|\frac{\mathrm{target}-\mathrm{average}(\mathrm{background})}{\mathrm{average}(\mathrm{background})}\right|\;$$

As shown in Table 2, the background suppression process assuredly enlarges the difference between the sub-pixel anomaly target and the background. The distance between the anomaly target and the background in the original RX detection result was nearly 3 times greater after performing one background suppression process, and this gap increased to more than 60 times by performing the background suppression process twice in this experiment.

For the second problem, as mentioned in Section 3.1, we rebuilt the spectral data based on each layer’s detection result. In theory, this process can suppress the spectral data whose result is small, but retain the spectral data whose result is large. Then, in the new rebuilt hyperspectral data cube, the anomaly target spectral data should be considerably different than the background spectral data. We analyze this theory from both an objective and subjective perspective. 

**Figure 8:**
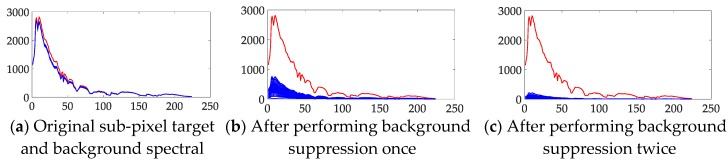
Sub-pixel anomaly target compared with background spectra. (**a**) Original sub-pixel target and background spectra; (**b**) these spectra after applying the background restrain process one time, and (**c**) these spectra after performing background suppression twice. **Vertical axis** stands for value of spectra vector, wheres **horizontal axis** stands for band numbers.

Subjectively, the spectra of the original data and the rebuilt data are compared directly in Figure 8. The original sub-pixel anomaly target spectra are similar to the background spectra; however, by using the restraint process, these spectra show greater differences in the rebuilt data. After two applications of the background suppression process, the target spectra remain unchanged and the background spectra have been further restrained. Then, after the background suppression, the target spectra remain unchanged and the background spectra are progressively suppressed.

To compare the difference between the sub-pixel anomaly target and the background spectra objectively, we introduce two spectral distance metrics to measure the distance among these spectra. The first distance metric we used is the spectral angle mapper (SAM) [28], which defines the shape difference between the spectra and is one of the most widely used spectral similarity metrics in hyperspectral classification and target detection tasks [29,30]. The second distance we used is the Euclidean distance [31], which provides a quantitative measure of the distance between two spectra vectors and is the one of the most widely used distance metrics in many feature matching applications.

The spectral difference between two spectra $x_{1}$ and $x_{2}$ with L bands can be analyzed as the calculated spectral dot product based on the Equation (30). The Euclidean distance metric between two spectra can be expressed the Equation (31).

(30) $$\theta {=\cos}^{-1}\left[\int x_{1}x_{2}dl/{\left[\int x_{1}{\left(l\right)}^{2}dl\right]}^{1/2}{\left[\int x_{2}{\left(l\right)}^{2}dl\right]}^{1/2}\right],$$

(31) $$D(x_{1},x_{2})=\left\|x_{1}-x_{2}\right\|=\sqrt{\sum_{l=1}^{L}{\left(x_{1}(l)-x_{2}(l)\right)}^{2}}.$$

We use the distance mentioned earlier to calculate the distance between the anomaly target and the average background spectra. In the ideal situation, the average background spectra may suppress the spectral vector close to the zero vector. To avoid this situation, we add a small positive value to each band value in the average background spectra. We set ε equal to 1, and then the average background spectra $x_{2}$ can be rebuilt, as shown in the following formula, and demonstrated in the results in Table 2.

(32) $$x_{2}=x_{2}(l)+\varepsilon \;$$

As shown in Table 3, the Euclidean distance and SAM score between the target spectra and the average background spectra are gradually enlarged in the rebuilt hyperspectral data. The Euclidean distance has increased by almost one order of magnitude, and the SAM score is almost 20 times greater after applying the data rebuilding process twice compared with the original hyperspectral data.

### 4.3. Sub-Pixel Anomaly Detection Experiments

We evaluate the proposed method using three hyperspectral datasets, including two pure ocean surface and one relatively complicated land cover scene. For the first two datasets, we use Target Implantation Method mentioned in [32] to generated sub-pixel anomaly targets by mixing clean ship target spectra and ocean background spectra with different abundance ratios, and then replaced them in the corresponding background pixels. This approach is somewhat different than the target implantation method mentioned in [33], which directly replaces the target and background spectra, whereas we mix them in varying proportions. To evaluate the detector’s performance more objectively, we add Gaussian white noise with 30-dB SNR in each of the mixed anomaly pixels. While this may not be the best method of generating the sub-pixel anomaly target, it is certainly better than replacing them directly.

For the third dataset, there already exists 21 pixels marked as anomaly targets, and thus we do not add any other synthetic targets in this scene. As we calculated with the linear unmixing method [34], the ratios of those targets ranges from 0.7 to 1, so, there are almost 8 sub-pixel anomalies (abundance ratios range from 0.7 to 0.9) and 13 full pixel anomalies (abundance ratios equals 1). Compared with the experiment on this dataset, the anomaly targets consist of different types of materials and different sizes. For example, the type in this dataset consists of embedded cars and roofs, where the size of the anomaly targets is one or two pixels. Furthermore, the observation area is more complex, with at least four types of objects constituting the background, which increases the difficulty to detect the anomaly targets.

**Figure 9:**
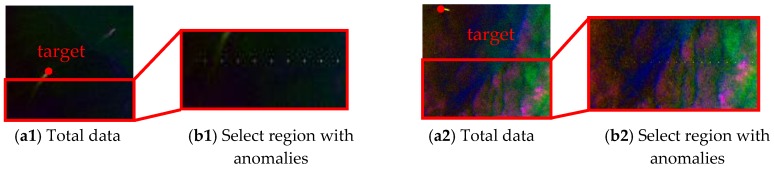

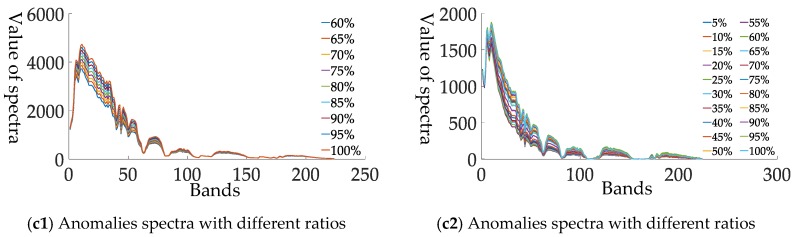
Total data, selected region and generated sub-pixel anomalies on Dataset-1 and Dataset-2. (**a1**,**a2**) Denote false color image of each data. (**b1**,**b2**) Denote false color image of the selected regions. (**c1**,**c2**) Denote generated sub-pixel anomalies with different abundance ratios.

**Figure 10:**
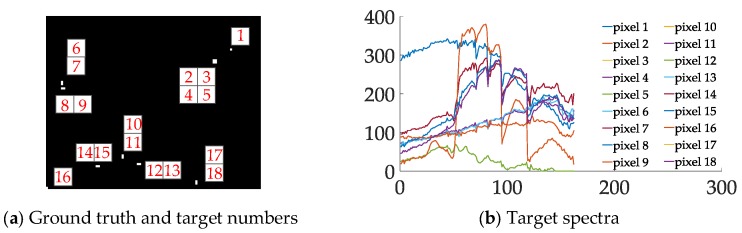
Targets spectra in Dataset-3. (**a**) Ground truth and target pixels’ numbers; (**b**) spectra of each pixel, vertical axis stands for value of spectra vector, horizontal axis stands for band numbers.

For space and brevity, only the patches involving the target are illustrated in the first two datasets (i.e., patch 7 in Dataset-1, patch 3 in Dataset-2). As shown in Figure 9, we generated sub-pixel anomalies with different abundance ratios (ranging from 0.6 to 1 in Dataset-1 patch 7 and 0.05 to 1 in Dataset-2 patch 3). For more details, the size of selected region is (35×100) and (100×200) and their upper-left locations are (66, 1) and (101, 1) respectively. In addition, the selected target locations are (57, 35) and (9, 32) in each dataset. For other patches, we implant the sub-pixel targets with the same process. Figure 10 illustrates the location of each target in Dataset-3. We draw corresponding spectra in Figure 10b.

**Figure 11:**
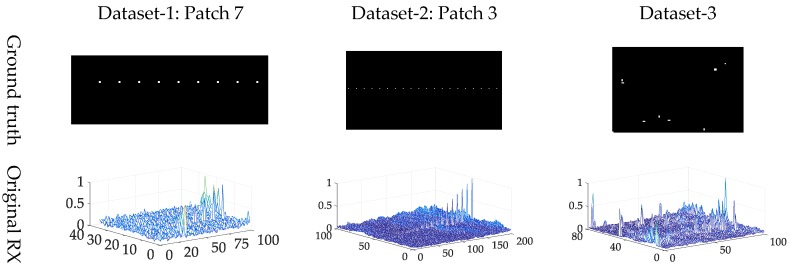

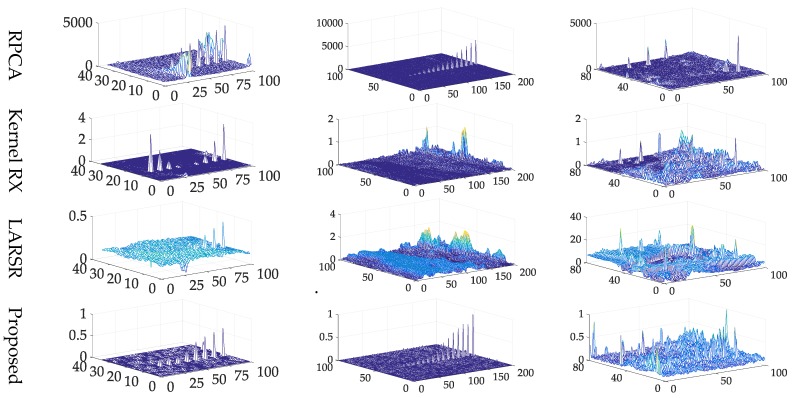
Experimental data on each dataset (Patch 7 in Dataset-1, Patch 3 in Dataset-2).

In the proposed method, two hyper parameters, including iteration numbers and the size of spatial regulation windows are illustrated in Table 4. The results of different anomaly target detection algorithms are listed in the Figure 11. For highlighting the anomalies, we use the 3-D figures to show the final results, because the 2-D figures use the grey index to show the score for each pixel and the differences of the backgrounds and anomalies are unclear in the view of 2-D figure. 

All the five methods have the ability to distinguish between anomalies and most background pixels on three datasets. But the proposed hierarchical method and Kernel RX show better visual performance than the other three algorithms. Considering the fact that the visual assessments may be inaccurate, we employ the semilog ROC curves and AUC score to further assess the detection performance.

**Figure 12:**
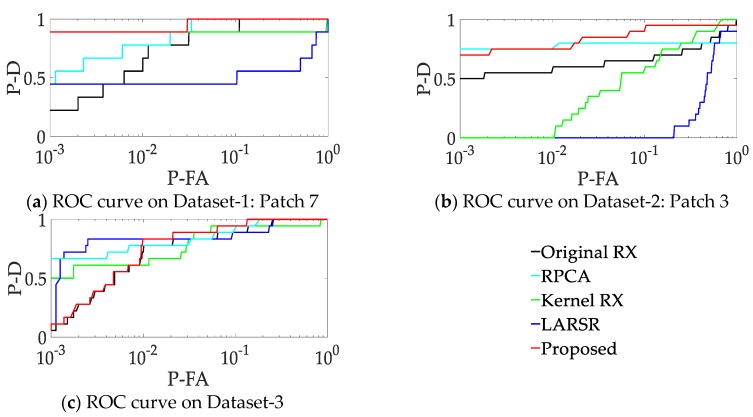
Semilog receiver operating characteristic (ROC) curves.

Figure 12 shows the semilog ROC curves of the five methods on the selected peaches. One can see that (1) For patch 7 of Dataset-1, the proposed method shows better performance than others. (2) For patch 3 of Dataset-2, RPCA and the proposed method show similar performance in low false alarm rate situation, but the latter is much better as false alarm rate growing. Moreover, with the increasing of false alarm rate, the unsatisfactory performance of Kernel RX enhances expeditiously. (3) For Dataset-3, the Kernel RX, LARSR and RPCA show better performance than the proposed method. But it is worth noting that the proposed method achieves the smallest false alarm rate when all the anomaly targets are detected.

In order to evaluate the performance in the low false alarm situation more clearly, we calculate the probability of detection when the false alarm rate equals 0.01. Additionally, we also calculate the false alarm rate when probability of detection equals 1. Here, we test all the patches and calculate the mean for the first two datasets. For the third dataset, we repeatly conduct the experiments 10 times, and then calculate the mean and standard deviation of the results. These resulting statistics are illustrated on Table 5.

We can see that the proposed method achieves the top two probability of detection (when false alarm rate equals 0.01) in all datasets. It proves that the proposed method has a good ability to distinguish most of anomalies with a low false alarm rate. When the probability of detection equals 1, the proposed method gets the second lowest false alarm rate to detect all the anomalies. Overall, our method shows better performance on detecting all the targets and getting higher probability of detection.

To further evaluate the efficiency of the proposed method, the AUC score and time consumption are listed from Table 6, Table 7, Table 8 and Table 9. Here, as all the methods show unsatisfactory performance on Dataset-2, we choose this dataset for detailed illustration in Table 6 and Table 7. Statistical (average and standard deviation) AUC score and time consumption from all the patches of the first two datasets and retest of the third dataset are illustrated in Table 8 and Table 9, respectively.

We test all the discussed methods on an Z820 Workstation with Intel Xeon E5-2630 and 128 GB RAM. The programming environment is MATLAB 2015a. Compared with the Original RX, the proposed hierarchical framework consists of different layers, which increases computational complexity to some extent. As illustrated in [35], the computational complexity of original RX is O(N), and thus, the computational complexity of the proposed method can be briefly calculated as:

(33) Complexity=O(i∗N),

where, i is the times of RX method employed in the proposed method. 

It can be seen that the proposed method achieves the highest average AUC score on all datasets as expected. Even though our method shows a little more time-consuming than the Original RX, it is still meaningful for some applications that need high detection accuracy and efficiency simultaneously. Further, our method not only get better detection results, but also much faster than other algorithms (i.e., RPCA, Kernel RX and LARSR).

It is worth noting that the performance of H-RX is close to other methods on Dataset-3 (i.e., similar AUC scores shown in Table 6). This results from the fact that the anomaly target could consist of more than one pixel (e.g., sub-pixel 2~5 in the Dataset-3). In this case, co-occurrences of multiple anomaly pixels within the spatial regularization window would perplex the judgement of true center anomaly one, and thus all of the sub-pixels may be restrained.

Finally, it is necessary to discuss unsatisfactory AUC scores of all the methods on the Dataset-2, which is caused by the low abundance ratios. For instance, when the abundance ratio is smaller than 0.25, the sub-pixel anomaly would be submerged in the background. Then, the H-RX fails to detect it with a low false alarm. Moreover, background is much more complex, especially for the background in the twelfth patch, which involves clouds, stripe noise and land cover regions. However, in such an extreme situation, the proposed method still outperforms the others in the detection results. Thus, the H-RX is a promising method for sub-pixel anomaly detection.

## 5. Conclusions

In this paper, we propose a hierarchical hyperspectral anomaly target detection method (H-RX) that consists of several layers with different functions. The main contributions of the proposed method are: (1) A novel hierarchical framework is proposed to restrain background spectra and enlarge the difference between the background and anomaly target spectra; (2) we prove the rationality of the proposed method theoretically and the ability to construct more discriminative spectra than that in previous layers; and (3) experimental results on three datasets show that the proposed hierarchical method has a significant improvement over the classical RX method and other recent sub-pixel anomaly detection methods.

## Figures and Tables

**Table 1:** Dataset information.

Dataset	Size	Bands	Resolution	Sub-Pixel Targets	Background
AVIRIS Dataset-1	(300×400)	224	15.5 m	Synthetic	Real-World
AVIRIS Dataset-2	(800×600)	224	15.5 m	Synthetic	Real-World
HYDICE Dataset-3	(80×100)	162	1 m	Real	Real-World

**Table 2:** Distance between target and background by using the nonlinear suppression function.

Algorithm	Original RX	Suppression-Once	Suppression-Twice
Distance	1.9	6.8	125.58

**Table 3:** Euclidean distance and SAM between sub-pixel anomaly and average background spectra.

Test Data	Euclidean Distance	SAM
Original data	$3.032\times 10^{3}$	0.0595
Rebuilt data after first layer	$1.124\times 10^{4}$	0.2073
Rebuilt data after second layer	$1.635\times 10^{4}$	0.9619

**Table 4:** Iterations and window size.

Dataset	Number of Iterations	Spatial Smooth Window
Dataset-1	2	3×3
Dataset-2	2	3×3
Dataset-3	1	5×5

**Table 5:** Anomaly detection results on three datasets. (P-D at P-FA = 0.01, P-FA at P-D = 1).

Algorithm	Dataset-1 (%)		Dataset-2 (%)		Dataset-3 (%)	
	P-D	P-FA	P-D	P-FA	P-D	P-FA
Original RX	72.3 ± 12.5	16.7 ± 7.6	53.7 ± 18.2	99.1 ± 2.6	76.8 ± 0.01	26.3 ± 0.01
RPCA	85.9 ± 9.2	9.7 ± 6.2	62.3 ± 24.8	100.0 ± 0.0	76.7 ± 0.00	17.8 ± 0.01
Kernel RX	85.0 ± 14.0	61.5 ± 45.64	29.2 ± 28.7	68.4 ± 22.9	61.3 ± 0.01	84.6 ± 0.02
LARSR	54.9 ± 31.9	81.6 ± 38.6	17.9 ± 35.2	95.2 ± 12.1	82.6 ± 0.01	26.9 ± 0.01
Proposed	89.1 ± 7.8	11.5 ± 7.5	65.4 ± 21.7	94.1 ± 12.9	83.4 ± 0.00	12.6 ± 0.00

**Table 6:** Area under the curve (AUC) score for each patches in Dataset-2.

Algorithm	Dataset-2 (%)											
	1	2	3	4	5	6	7	8	9	10	11	12
Original RX	79.9	87.9	80.7	90.9	80.6	77.4	87.1	78.7	49.6	85.8	85.6	30.8
RPCA	54.0	77.7	74.9	74.9	74.4	19.6	79.8	70.0	0.0	74.7	78.8	0.0
Kernel RX	70.0	84.6	83.5	90.4	81.5	67.1	88.4	87.9	61.2	86.3	84.3	50.8
LARSR	50.8	65.9	46.9	56.3	55.8	54.8	73.5	57.9	63.0	99.4	56.6	47.0
Proposed	94.7	94.9	88.0	89.8	94.9	89.6	89.5	90.0	84.4	95.0	95.0	54.5

**Table 7:** Time consumption performances each patches in Dataset-2.

Algorithm	Dataset-2 (s)											
	1	2	3	4	5	6	7	8	9	10	11	12
Original RX	4.8	4.8	4.3	4.7	4.8	4.6	4.7	4.2	4.8	4.3	4.8	4.6
RPCA	70.7	57.7	56.6	121.2	59.0	48.4	73.7	62.1	100.4	56.9	58.8	157.9
Kernel RX	755.35	818.68	1087.3	2962.91	811.7	876.6	1521.7	919.2	768.4	1478.9	683.5	801.1
LARSR	9.1	9.9	9.9	11.6	9.7	7.9	10.6	8.7	7.9	6.3	10.3	6.8
Proposed	5.7	5.3	4.8	5.4	5.3	5.4	5.2	4.7	5.3	4.7	5.2	5.2

**Table 8:** AUC score (For Dataset-1 and Dataset-2, we calculate the average AUC and standard deviation of each patch).

Algorithm	AUC Score (%)		
	Dataset-1	Dataset-2	Dataset-3
Original RX	92.70 ± 2.2	76.25 ± 17.79	96.98 ± 0.01
RPCA	96.58 ± 1.8	56.57 ± 31.27	97.42 ± 0.01
Kernel RX	92.81 ± 6.6	78.01 ± 12.69	94.21 ± 0.01
LARSR	46.29 ± 30.6	60.66 ± 14.37	96.62 ± 0.00
Proposed	97.71 ± 1.7	88.36 ± 11.22	98.39 ± 0.00

**Table 9:** Time consumption performances (For Dataset-1 and Dataset-2, we calculate the average and standard deviation of each patch.).

Algorithm	Average Time Consumption (s)		
	Dataset-1	Dataset-2	Dataset-3
Original RX	0.79 ± 0.02	4.61 ± 0.23	0.88 ± 0.04
RPCA	51.08 ± 5.90	76.96 ± 33.01	139.11 ± 1.61
Kernel RX	13.34 ± 5.81	1123.26 ± 641.14	83.96 ± 1.17
LARSR	1.61 ± 0.37	9.06 ± 1.59	3.08 ± 0.29
Proposed	1.29 ± 0.11	6.17 ± 0.31	1.25 ± 0.13

## References

[1] Mo C., Kim G., Lim J., Kim M.S., Cho H., Cho B.K. (2015). Detection of Lettuce Discoloration Using Hyperspectral Reflectance Imaging. Sensors.

[2] Lacar F.M., Lewis M.M., Grierson I.T. Use of hyperspectral imagery for mapping grape varieties in the Barossa Valley, South Australia. Proceedings of the International Geoscience and Remote Sensing Symposium, Scanning the Present and Resolving the Future.

[3] Meer F.V.D. (2004). Analysis of spectral absorption features in hyperspectral imagery. Int. J. Appl. Earth Obs. Geoinf..

[4] Malthus T., Mumby P. (2003). Remote sensing of the coastal zone: An overview and priorities for future research. Int. J. Remote Sens..

[5] Han Y., Li J., Zhang Y., Hong Z., Wang J. (2017). Sea Ice Detection Based on an Improved Similarity Measurement Method Using Hyperspectral Data. Sensors.

[6] Bioucas-Dias J.M., Plaza A., Camps-Valls G., Scheunders P., Nasrabadi N., Chanussot J. (2013). Hyperspectral Remote Sensing Data Analysis and Future Challenges. IEEE Geosci. Remote Sens. Mag..

[7] Matteoli S., Diani M., Corsini G. (2010). A tutorial overview of anomaly detection in hyperspectral images. Aerosp. Electron. Syst. Mag..

[8] Reed I.S., Yu X. (1990). Adaptive multiple-band CFAR detection of an optical pattern with unknown spectral distribution. IEEE Trans. Acoust. Speech Signal Process..

[9] Wright J., Ganesh A., Rao S., Ma Y. Robust Principal Component Analysis: Exact Recovery of Corrupted Low-Rank Matrices. Proceedings of the 23rd Annual Conference on Advances in Neural Information Processing Systems.

[10] Xu Y., Wu Z., Li J., Plaza A., Wei Z. (2016). Anomaly Detection in Hyperspectral Images Based on Low-Rank and Sparse Representation. IEEE Trans. Geosci. Remote Sens..

[11] Zhang Y., Du B., Zhang L., Wang S. (2016). A Low-Rank and Sparse Matrix Decomposition-Based Mahalanobis Distance Method for Hyperspectral Anomaly Detection. IEEE Trans. Geosci. Remote Sens..

[12] Borghys D., Kåsen I., Achard V., Perneel C. (2012). Comparative evaluation of hyperspectral anomaly detectors in different types of background. Algorithms and Technologies for Multispectral, Hyperspectral, and Ultraspectral Imagery XVIII.

[13] Nasrabadi N.M., Shen S.S., Lewis P.E. (2008). Regularization for spectral matched filter and RX anomaly detector—Art. no. 696604. Algorithms and Technologies for Multispectral, Hyperspectral, and Ultraspectral Imagery Xiv.

[14] Kwon H., Nasrabadi N.M. (2005). Kernel RX-algorithm: A nonlinear anomaly detector for hyperspectral imagery. IEEE Trans. Geosci. Remote Sens..

[15] Zhao C., Deng W., Yan Y., Yao X. (2017). Progressive Line Processing of Kernel RX Anomaly Detection Algorithm for Hyperspectral Imagery. Sensors.

[16] Williams J.P., Bihl T.J., Bauer K.W. (2013). Towards the mitigation of correlation effects in anomaly detection for hyperspectral imagery. J. Defense Model. Simul..

[17] Williams J., Bihl T., Bauer K. (2010). Mitigation of Correlation and Heterogeneity Effects in Hyperspectral Data. Intell. Eng. Syst. Through Artif. Neural Netw..

[18] Gao C., Meng D., Yang Y., Wang Y., Zhou X., Hauptmann A.G. (2013). Infrared Patch-Image Model for Small Target Detection in a Single Image. IEEE Trans. Image Process..

[19] Anderson K.L., Iltis R.A. (1997). A tracking algorithm for infrared images based on reduced sufficient statistics. IEEE Trans. Aerosp. Electron. Syst..

[20] Xiong Y., Peng J.X., Ding M.Y., Xue D.H. (1997). An extended track-before-detect algorithm for infrared target detection. IEEE Trans. Aerosp. Electron. Syst..

[21] Manolakis D., Marden D., Shaw G.A. (2003). Hyperspectral Image Processing for Automatic Target Detection Applications. Linc. Lab. J..

[22] Chang C.I. (2010). Multiparameter Receiver Operating Characteristic Analysis for Signal Detection and Classification. IEEE Sens. J..

[23] Clare P.E., Bernhardt M., Oxford W.J. (2003). A new approach to anomaly detection in hyperspectral images. Proc. SPIE.

[24] Rakotomamonjy A. Optimizing area under ROC curve with SVMs. Proceedings of the 1th International Workshop on ROC Analysis in Artificial Intelligence (ROCAI).

[25] Green R.O., Chrien T.G., Enmark H.T. (1987). First Results from the Airborne Visible/Infrared Imaging Spectrometer (AVIRIS). Remote Sens. Environ..

[26] Porter W.M., Enmark H.T. (1988). A System Overview of the Airborne Visible/Infrared Imaging Spectrometer (Aviris). Proc. SPIE.

[27] Mitchell P.A. Hyperspectral digital imagery collection experiment (HYDICE). Proceedings of the Geographic Information Systems, Photogrammetry, and Geological/Geophysical Remote Sensing.

[28] Kruse F.A., Lefkoff A.B., Boardman J.W., Heidebrecht K.B., Shapiro A.T., Barloon P.J., Goetz A.F.H. (1993). The spectral image processing system (SIPS)-interactive visualization and analysis of imaging spectrometer data. Remote Sens. Environ..

[29] Rao N.R., Garg P.K., Ghosh S.K. (2007). Development of an agricultural crops spectral library and classification of crops at cultivar level using hyperspectral data. Precis. Agric..

[30] Nidamanuri R.R., Zbell B. (2010). A method for selecting optimal spectral resolution and comparison metric for material mapping by spectral library search. Progr. Phys. Geogr..

[31] Keshava N. (2004). Distance metrics and band selection in hyperspectral processing with applications to material identification and spectral libraries. IEEE Trans. Geosci. Remote Sens..

[32] Stefanou M.S., Kerekes J.P. (2009). A Method for Assessing Spectral Image Utility. IEEE Trans. Geosci. Remote Sens..

[33] Ren H., Chang C., Rand R.S. (2008). How to design synthetic images to validate and evaluate hyperspectral imaging algorithms. Algorithms and Technologies for Multispectral, Hyperspectral, and Ultraspectral Imagery XIV.

[34] Bioucas-Dias J.M. A variable splitting augmented Lagrangian approach to linear spectral unmixing. Proceedings of the 2009 Workshop on Hyperspectral Image & Signal Processing: Evolution in Remote Sensing.

[35] Chen S.Y., Wang Y., Wu C.C., Liu C. (2014). Real-time causal processing of anomaly detection for hyperspectral imagery. Aerosp. Electron. Syst. IEEE Trans..

